# First Description of the Mitogenome and Phylogeny of Culicinae Species from the Amazon Region

**DOI:** 10.3390/genes12121983

**Published:** 2021-12-14

**Authors:** Bruna Laís Sena do Nascimento, Fábio Silva da Silva, Joaquim Pinto Nunes-Neto, Daniele Barbosa de Almeida Medeiros, Ana Cecília Ribeiro Cruz, Sandro Patroca da Silva, Lucas Henrique da Silva e Silva, Hamilton Antônio de Oliveira Monteiro, Daniel Damous Dias, Durval Bertram Rodrigues Vieira, José Wilson Rosa, Roberto Carlos Feitosa Brandão, Jannifer Oliveira Chiang, Livia Carício Martins, Pedro Fernando da Costa Vasconcelos

**Affiliations:** 1 Department of Arbovirology and Hemorrhagic Fevers, Ananindeua, Evandro Chagas Institute—IEC/SVS/MS, Belém 67030-000, Brazil; brunanascimento@iec.gov.br (B.L.S.d.N.); fabiodasilva@iec.gov.br (F.S.d.S.); danielemedeiros@iec.gov.br (D.B.d.A.M.); anacecilia@iec.gov.br (A.C.R.C.); sandrosilva@iec.gov.br (S.P.d.S.); hamiltonmonteiro@iec.gov.br (H.A.d.O.M.); durvalvieira@iec.gov.br (D.B.R.V.); josejr@iec.gov.br (J.W.R.J.); robertobrandao@iec.gov.br (R.C.F.B.); janniferchiang@iec.gov.br (J.O.C.); liviamartins@iec.gov.br (L.C.M.); 2 Biological and Health Sciences Center, Parasitary Biology in the Amazon Posgraduation Program, State of Pará University, Belém 66095-662, Brazil; lucashenriqueuepa@gmail.com (L.H.d.S.e.S.); damous1994@gmail.com (D.D.D.); pedrovasconcelos@iec.gov.br (P.F.d.C.V.)

**Keywords:** Culicidae, mitochondrial genome, sequencing, molecular taxonomy

## Abstract

The Culicidae family is distributed worldwide and comprises about 3587 species subdivided into the subfamilies Anophelinae and Culicinae. This is the first description of complete mitochondrial DNA sequences from *Aedes fluviatilis*, *Aedeomyia squamipennis*, *Coquillettidia nigricans*, *Psorophora albipes*, and *Psorophora ferox.* The mitogenomes showed an average length of 15,046 pb and 78.02% AT content, comprising 37 functional subunits (13 protein coding genes, 22 tRNAs, and two rRNAs). The most common start codons were ATT/ATG, and TAA was the stop codon for all PCGs. The tRNAs had the typical leaf clover structure, except *tRNA^Ser1^*. Phylogeny was inferred by analyzing the 13 PCGs concatenated nucleotide sequences of 48 mitogenomes. Maximum likelihood and Bayesian inference analysis placed *Ps. albipes* and *Ps. ferox* in the *Janthinosoma* group, like the accepted classification of *Psorophora* genus. *Ae. fluviatilis* was placed in the Aedini tribe, but was revealed to be more related to the *Haemagogus* genus, a result that may have been hampered by the poor sampling of *Aedes* sequences. *Cq. nigricans* clustered with *Cq. chrysonotum*, both related to *Mansonia*. *Ae. squamipennis* was placed as the most external lineage of the Culicinae subfamily. The yielded topology supports the concept of monophyly of all groups and ratifies the current taxonomic classification.

## 1. Introduction

The Family Culicidae (Meigen, 1818), known as mosquitoes, is a large taxon distributed worldwide. The Culicidae comprises about 3587 officially recognized species, classified in the subfamilies Anophelinae and Culicinae, accepted to be monophyletic despite some inner relations that are not entirely understood [1]. This family of insects is responsible for transmitting the etiologic agents of several arboviruses such as dengue virus, yellow fever virus, Chikungunya virus, and Zika virus, filariosis from *Wuchereria bancrofti* and malaria, caused by *Plasmodium* spp. [2].

The Subfamily Anophelinae includes the genera *Anopheles*, *Bironella* and *Chagasia*, while the subfamily Culicinae includes a variety of genera within the tribes Aedeomyiini, Aedini, Culicini, Culisetini, Ficalbiini, Hoggesiini, Mansoniini, Orthopodomyiini, Sabethini, Toxorhynchitini, and Uranotaeniini, totalizing 110 genera [1,2,3,4]. The Anophelinae subfamily is demonstrably monophyletic, a basal lineage in relation to the Culicidae family. To date, 493 species are recorded, with many complex and sibling-species relationships awaiting deeper phylogenetic and taxonomic resolution, mainly in the *Anopheles* genus [1,5,6]. The unknown medical/veterinary importance of *Bironella* and *Chagasia* makes most of the studies focus towards the genus *Anopheles* due its capacity to transmit malaria parasites, in addition to several arboviruses [7,8]. The monophyletic status for *Bironella* is not supported in most of the studies [6,9], but the opposite is found to the genus *Anopheles*, specifically the subgenus *Stethomyia*, *Lophopodomyia*, *Kerteszia* and *Cellia* [9].

Like the Anophelinae, the phylogenetic history of the Culicinae subfamily is not completely settled, but some groups have been better studied due their epidemiological role as potential vectors of microorganisms. Former studies in the Sabethini tribe had investigated its phylogenetic relationships mainly through morphological and biogeographical aspects, and despite the fact the Sabethini was considered to be monophyletic, such cladistics analysis displayed some divergences among the genera, notably in the *Wyeomyia* genus recovered as a large paraphyletic taxa [2,10]. More recent mitogenome studies reiterated the monophyly of neotropical Sabethini species corroborating Reidenbach’s findings based on both morphological and molecular data [11,12]. The Culicini tribe has grounded monophyletic status, but there are many cryptic species that hamper the natural classification of the group, mainly in the *Culex* genus [13,14,15]. The Aedini tribe is undoubtedly the most critical group in terms of phylogenetic reconstruction, it is an enormous group where over time, dozens of subgenera rose to genera status followed by restoration, and until now there is no phylogeny consensus. In a broader view, the tribe is treated as monophyletic and encompassing three genera (traditional classification: *Aedes*, *Haemagogus* and *Psorophora*), but its interrelations lack deeper and wider analysis, especially for the genus *Aedes* [1,2,3,4].

In order to improve taxonomic and evolutionary studies, the use of genomic tools has increasingly contributed significant information, for example, for the development of vector control strategies or population variability assessments [16,17,18]. In this context, one of the genomic markers most currently used is the mitochondrial genome (mitogenome), which consists of a compact double-stranded circular molecule, with a length ranging from 15 to 20 kb, housing 37 functional subunits, 13 of which are active in protein coding (PCGs), 22 regions of transporter RNA (tRNA) and two ribosomal RNA regions, in addition to a terminal region rich in adenine and thymine (A+T), associated with replicative processes of the molecule [19]. The use of this genomic set is considered advantageous for the absence of introns, the relative low gene recombination and for its maternal haploid inheritance. For the past few years, the use of the mitogenome has been producing relevant results in the development of evolutionary investigations for a wide variety of groups of organisms, ranging from insects to mammals, but in particular arthropods of medical-epidemiological importance [5,20,21,22,23,24,25,26].

Despite the great number of Culicidae of medical importance, particularly in the context of the Brazilian Amazon region, there is in contrast no availability of genomic information in public data repositories of these organisms with few exceptions, and considering the great potential of the applicability of molecular markers such as the mitochondrial genome in the development of investigations of mosquito molecular taxonomy, we present in this study, for the first time, the characterization of the mitogenome of the species *Aedeomyia squamipennis* Lynch Arribálzaga, 1878, *Aedes* (*Grc.*) *fluviatilis* Lutz, 1904, *Coquillettidia* (*Rhy.*) *nigricans* Coquillet, 1904, *Psorophora* (*Jan*.) *albipes* Theobald, 1907, and *Psorophora* (*Jan.*) *ferox* Humboldt, 1819, all occurring in the Brazilian Amazon, using high throughput sequencing (HTS) and evolutionary considerations. 

## 2. Materials and Methods

### 2.1. Sample Collection and Total DNA Extraction

The mosquito species investigated in this study derived from several ecoepidemiological investigations realized by the Department of Arbovirology and Haemorrhagic Fevers of Evandro Chagas Institute (IEC/SVS/MS). Sample collection were performed by the human attraction method during field expeditions in the municipalities of Altamira, Canaã dos Carajás, Santa Bárbara and Belém (Combu Island), in the state of Pará, carried out between 2017 and 2019 (Table 1).

The authorization to carry out activities allowing the collection of biological samples of mosquitoes was granted by the Biodiversity Information and Authorization System of the Ministry of Environment (SISBIO/MMA) under registration numbers 56004-1 and 56504-5. The specimens collected were identified based on external morphology characteristics with dichotomous keys for representatives of the subfamily Culicinae [4], later the identification was confirmed by comparison with DNA barcode sequences available on GenBank (Table 2). The specimens were organized in groups containing up to 45 individuals belonging to the same species, following storage at −70 °C for further procedures. Following the taxonomic identification process, five mosquitoes were removed from each species-group to compose the biological sample to be sequenced. The samples were macerated individually (per species) using 3 mm stainless steel spheres in a TissueLyser II device (Qiagen, Hilden, ME, Germany), and soon after, total DNA was extracted using a commercial DNeasy Blood & Tissue kit (Qiagen, Hilden, ME, Germany), following the recommendations of the manufacturer.

### 2.2. Quantification of Total Extracted DNA, Construction of the Genomic Library, and Sequencing

The total DNA extracted from each lot of identified species was quantified using the Qubit 4.0 fluorometer equipment (Life Technologies, Carlsbad, CA, USA) with the commercial Qubit dsDNA Hs Assay kit (Invitrogen, Waltham, MA, USA), following the recommendations of the manufacturers. The total DNA products extracted from each batch were previously standardized at a concentration of 0.2 ng/µL, and then fragmented and labeled with adapter sequences (i7 and i5) following the recommendations of the Nextera XT DNA library preparation (Illumina, San Diego, CA, USA) protocol. The labeled DNA fragments were then purified using the Agencourt AMPure XP kit (Beckman Coulter, Brea, CA, USA). After completion of the previous steps, the genomic libraries obtained were quantified using the Qubit 4.0 fluorometer (Life Technologies, Carlsbad, CA, USA), and their quality was checked using a High Sensitivity DNA kit (Agilent Technologies, Santa Clara, CA, USA), following the recommendations established by the manufacturer. Finally, the products obtained were sequenced using the NextSeq 500/550 High Output Kit and in a run of 150 cycles (2 × 75) NextSeq500 platform (Illumina, San Diego, CA, USA).

### 2.3. Pre-Processing of Sequenced Products

At the end of the genomic sequencing reaction, the data generated in Illumina Base Call (.bcl) format were transferred from the NextSeq 500/550 platform equipment to a computer workstation, where the analysis and genomic characterization steps were performed. Files in “.bcl” format were converted to “.fastq” format using bcl2fastq Conversion v.2.20 (Illumina, San Diego, CA, USA) software. The FastQC v.0.11.9 software [27] was used to previously verify the quality metrics of the data obtained, and then, the software Trim Galore v.0.6.5 [28] was used for the removal of adapter sequences. At the end of this process, a new quality check and validation of the data obtained was performed using FastQC.

### 2.4. Genomic Assembly

The pre-processed sequencing files of each species investigated in this study were submitted to a genomic assembly step by the de novo method using the software MEGAHIT v.1.2.9 [29] and SPAdes v.3.15.2 [30]. The contigs generated and corresponding to the mitochondrial readings of each species evaluated were selected using the DIAMOND software [31], and visualized using the MEGAN6 software [32]. The assembled contigs corresponding to the mitochondrial genome of each species were compared to genomic data from reference sequences (*Aedes albopictus* Genbank ID: NC_006817, and *Coquillettidia chrysonotum* Genbank ID: NC_044655), and were manually inspected using Geneious v.11.0 software [33].

### 2.5. Analyses of the Obtained Mitochondrial Sequences

The online MITOchondrial genome annotation Server (MITOS—http://mitos.bioinf.uni-leipzig.de/index.py, accessed on 1 November 2021) tool [34] was used for automatic annotation and prediction of secondary tRNA structures of the mitochondrial sequences obtained in this study. The graphic maps containing the representations of the mitochondrial genomes obtained were generated using the CGView software [35], and the nucleotide composition metrics of each sequence were obtained using the Geneious v.11.0 and MEGAX software [36]. Based on the nucleotide composition metrics obtained, the composition biases based on the AT and GC asymmetry values were estimated using the formulas: AT-skew = (A% − T%)/(A% + T%) and GC-skew = (G% − C%)/(G% + C%) [37]. In addition, the relative use of synonymous codons (RSCU) was estimated based on the set of all 13 PCGs, using MEGAX. A sliding window of 200 bp in steps 25 bp was performed using the PopGenome package [38] in the statistical software R [39], and the pairwise comparison of the proportions of non-synonymous (*dN*) and synonymous (*dS*) substitutions (*dN*/*dS*) among the obtained sequences were calculated using the CodeML software, part of the PAML package [40]. For plotting in graphs the results obtained based on nucleotide composition metrics, AT/GC skews, RSCU biases, and *dN*/*dS* ratios, the statistical software R was used (R Foundation for Statistical Computing, Vienna, Austria. Available in: https://www.R-project.org/, accessed on 1 November 2021).

### 2.6. Phylogenetics Analysis

Using the Geneious v.11.0 software, all 13 PCGs of each of the sequences obtained in this study were extracted together, as well as other taxa available in the Genbank-NCBI and EMBL data repositories, being aligned using the MAFFT algorithm [41]. Nucleotide distances between the sequences used were obtained using the MEGAX software, based on the maximum composite likelihood method. The reconstruction of the phylogenetic relationships between the analyzed species was based on two different methodologies: for the definition of the best nucleotide substitution model according (having been defined the GTR+F+R4 model) to the Akaike information criterion (AIC) and the phylogeny reconstruction using the maximum likelihood methodology, the IQtree v.1.6.12 software [42] was used with bootstraping values defined for 1000 repetitions; and for the reconstruction of the phylogeny using the Bayesian inference methodology, the MrBayes v3.2.7a software [43] was used, with the performance of two simultaneous and independent reactions, with four chains each (three hot chains and a cold chain), defined for 1,000,000 generations, with sampling of the topologies generated every 1000 steps, and with a relative burn of 25%. Finally, the obtained topologies were visualized using Figtree v.1.4.4 software [44], and edited using Inkscape [45].

## 3. Results and Discussion

### 3.1. Organization, Structure and Content of the Mitogenomes Obtained

**Figure 1:**
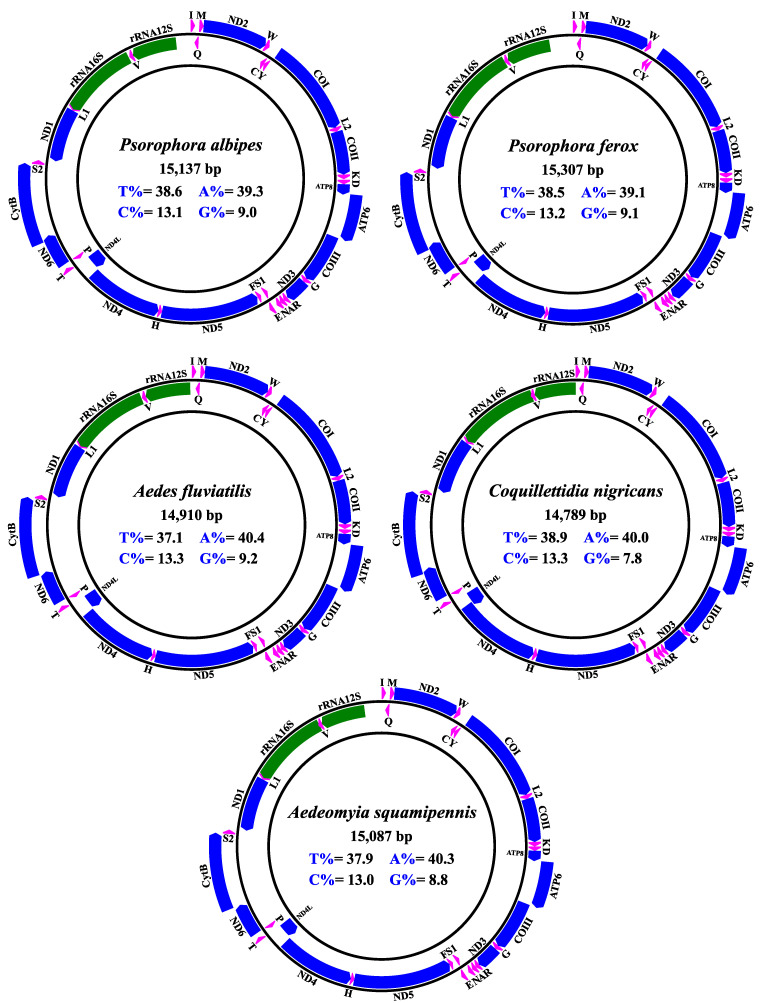
Structural representations of the *Ps. albipes*, *Ps. ferox*, *Ae. fluviatilis*, *Cq. nigricans* and *Ad. squamipennis* mitogenomes. The internal values indicate the overall content of the nucleotide bases. The blue, pink, and green blocks indicate PCGs, tRNAs, and rRNAs, respectively. Each tRNA is identified by a unique letter abbreviation. The genes arranged in the outer circle are located in the J strand (Forward), and the genes arranged in the inner circle are located in the N strand (Reverse).

The mitogenome of *Ad. squamipennis*, *Cq. nigricans*, *Ae. fluviatilis*, *Ps. albipes*, and *Ps. ferox* (Tables S1–S5), were arranged into classical compact circular double-strand DNA molecules, counting 37 functional and conserved subunits: 13 PCGs, 22 tRNAs, and two rRNA, organized along the J (forward) and R (reverse) strands (Figure 1).

**Figure 2:**
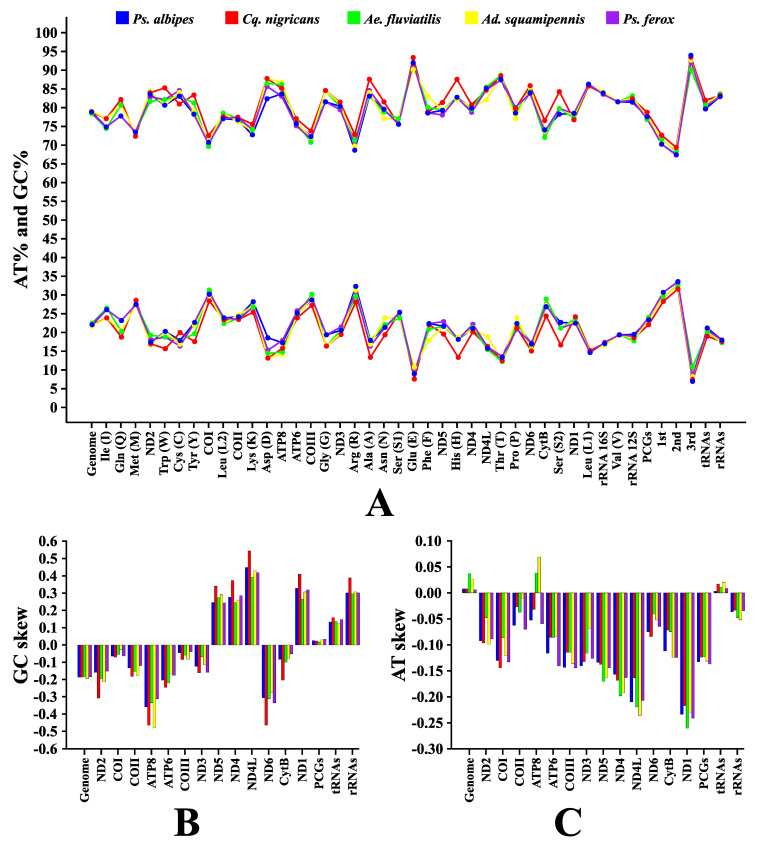
Information on AT/GC contents, and AT/GC-skews of the investigated mitogenomes. (**A**) AT contents (%). (**B**) AT-skews. (**C**) GC-skews.

The size of the sequences obtained ranged from 14,789 bp (*Cq. nigricans*) to 15,307 bp (*Ps. ferox*), and the overall AT content ranged from 77.5% (*Ae fluviatilis*) to 78.9% (*Cq. nigricans*). Considering the sets of PCGs, tRNAs and rRNAs, the species presented, respectively, average AT compositions of 76.6%, 79.7%, and 82.3% (Figure 2A, Table 3). These results are similar to the putative characteristics from previous reported mosquito mitogenomes. The mitogenome of insects in general displays some particular features like the highly conserved genome architecture, low GC content and codon usage bias, such characteristics were recorded in this study [5,19,22,23,46].

Additionally, the analysis of the composition asymmetry for each mitogenome resulted in positive values for AT-skews and negative values for CG-skews (Figure 2B,C, Table 3), meaning the greater proportion of adenine and cytosine on the majority chain compared to the thymine and guanine ratio. These findings resemble the results of previous studies that observed a nucleotide composition pattern for Culicidae [5,22,23,46,47,48], and other insect groups [24,26,49,50].

All five of the mitogenomes presented a set of 22 tRNAs each. The classical cloverleaf-like structure, with four arms [amino acid acceptor (AA), dihydrouridine (DHU), TΨC, and anticodons (AC)] and loops [AA, DHU, TΨC, and variable (V)] were retrieved by secondary structure prediction analysis from 21 of the 22 subunits. The remaining unit *tRNA^Ser1^* shows a notable feature of reduced DHU arm, replaced by a loop. Same features reported in earlier research with Culicidae and other insects [5,22,23,46,47,49,50,51,52]. The tRNAs had an average length of 1484 bp and average overall AT content of 79.7%. Furthermore, 15 mismatched pair bases were detected for *Ae. fluviatilis* (13 GU and two UU pairs), 19 mismatches were identified for *Ad. squamipennis* (16 GU and three UU pairs), 20 mismatches for *Ps. ferox* (19 GU and one UU), 24 mismatches recorded for both *Cq. nigricans* (21 GU and three UU), and *Ps. albipes* (23 GU and one UU) (Figures S1–S5).

Regarding only the PCGs subunits, the lengths ranged from 11,170 bp of *Ad. squamipennis* (AT% content of 76.7%) to 11,357 bp of *Cq. nigricans* (AT% content of 77.8%). All PCGs had negative values for AT-skews, except for subunit *ATP8* in *Ae. fluviatilis* and *Ad. squamipennis* (Figure 2B). Likewise, GC-skews were often negative, except for the subunits *ND5*, *ND4*, *ND4L* and *ND1* for all sequences (Figure 2C). All the PCGs stop codon for the five species was TAA and the most frequent start codon were ATT/ATG. The AT content increased substantially in the third position, up to 93% in *Ps. albipes* (in *Cq. nigricans*, 92.5%; *in Ad. squamipennis*, 91.7%; in *Ps. ferox*, 91.4%; and in *Ae. fluviatilis*, 89.2%) (Tables S1–S5).

The subunits recognized as the putative *rRNA*^16S^ and *rRNA*^12S^ when concatenated ranged from 2097 bp (*Cq. nigricans*) to 2160 (*Ad. squamipennis* and *Ps. ferox*). The AT content were actually very similar, despite almost all species being from different genera (average of 82.4%), also, in the five mitogenomes obtained global AT-skews were negative and GC-skews positive (Figure 2B,C). Individually, the larger *rRNA*^16S^ counted 1376 bp (*Cq. nigricans*) and the shorter 1359 bp (*Ps. albipes*). For *rRNA*^12S^ the larger subunit counted 795 bp (*Ps. albipes* and *Ps. ferox*) and the shorter 721 bp (*Cq. nigricans*). As already reported previously, in the five sequences the subunit *rRNA*^16S^ was flanked by the *t*RNA*^Leu1^* and *t*RNA*^Val^* and *rRNA*^12S^ was flanked by the same *t*RNA*^Val^* and the control region, the last non-sequenced. Such values are in agreement with earlier mosquito mitogenome studies [5,22,23,48].

The sequences obtained presented between eight and 15 small intergenic and non-coding regions, with lengths ranging from one bp to 34 bp, thus totaling in *Ps. albipes* 105 non-coding bp; in *Cq. nigricans*, 108 bp; in *Ae. fluviatilis*, 124 bp; in *Ad. squamipennis*, 65 bp; and in *Ps. ferox*, 101 bp. Previous studies related such small intergenic spaces, and it was thought to be related to occurrence of overlapping reading frames and accordingly compact genome structure, such as the mitogenome [23,53].

No data for A+T region control were obtained in this study from any of the five species. As broadly known, this region is naturally rich in homopolymers, which constitutes an impairment for next generation sequencers. Furthermore, in invertebrates this particular region is highly variable in terms of length and rates of mutations (significant and non-significant). Similar to the presented results, this region was not obtained in other studies either. Lemos et al. [54] and Silva et al. [23], in their studies on the characterization of the mitogenome of species of the genus *Haemagogus*, did not recover this region from the obtained sequences. In order to improve the sequencing assays, alternative methods can be used to fully fill this lacuna such as conventional PCR and Sanger’s sequencing method, once they allow target amplification, as already proved in similar studies [55,56,57,58].

### 3.2. Description of Protein-Coding Genes (PCGs)

For all the five mitogenomes obtained, nine PCGs displayed sense of transcription on the forward strand (J): *ATP6*, *ATP8*, *COI*, *COII*, *COIII*, *CytB*, *ND2*, *ND3*, and *ND6*, and the remaining genes showed sense of transcription on the reverse strand (N): *ND5*, *ND4*, *NDL4*, and *ND1.* A variety of start codons were used, present in the five sequences: ATT, ATG, TTG, and GTG (except in *Cq. nigricans*). Other start codons were also detected such as ATC (*Ae. fluviatilis*, *Cq. nigricans*, and *Ps. ferox*) and ATA (*Ad. squamipennis*), and all genes used TAA as standard stop codon (Tables S1–S5). Same arrangement and composition were previously described in other studies with Culicidae mitogenome [5,22,23,47].

In the genes *ND2*, *COI*, and *ND3*, for all species the start codon recorded was ATT, the same start codon is displayed in *ATP8*, *ND6*, and *CytB* from *Ps. albipes*; also for *ND4* and *CytB* from *Cq. nigricans*; *ND3* and *CytB* from *Ae. fluviatilis*, *ATP8* and *ND6* from *Ad. squamipennis*; and *ATP8* and *CytB* from *Ps. ferox* sequence. The ATG start codon was recorded from *COIII* and *ND4L* for all species, additionally this codon also signals encoding for *COII* and *ND4* from *Ps. albipes* and *Ps. ferox*, *COII* and *ND5* from *Cq. nigricans*, *COII* and *ATP6* from *Ae. fluviatillis* and *ATP6*, *ND4* and *CytB* from *Ad. squamipennis* mitogenome. The TTG start codon was retrieved from the *ND1* gene for all species, and individually for *ATP6* from *Cq. nigricans*, *Ps. albipes*, and *Ps. ferox*. The GTG start codon signaling process for the ND5 gene in all species, except for *Cq. nigricans*. The ATC start codon was recorded for *ATP8* and *ND6* for both *Cq. nigricans* and *Ae. fluviatilis*, and *ND6* from *Ps. ferox*, and finally the ATA start codon was recorded only for *COII* from *Ad. squamipennis*. No incomplete stop codons(T/TA) were found in any of the sequences (Tables S1–S5), although this is a relatively common event in Culicidae and other insects [22,23,46,47].

**Figure 3:**
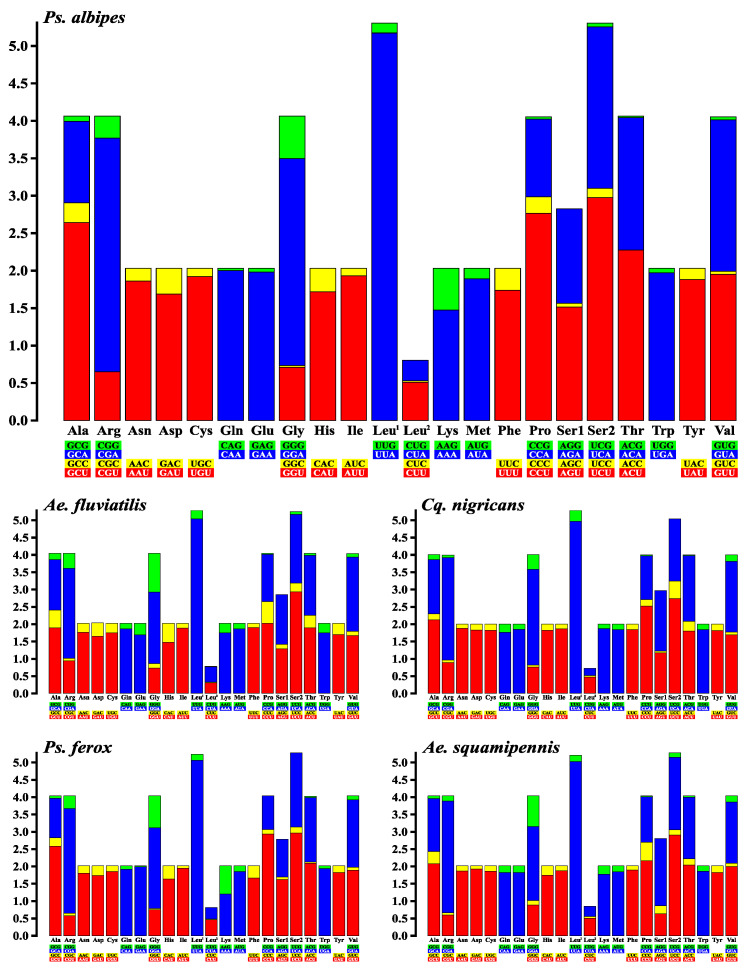
Relative synonymous codon usage (RSCU) of the obtained mitogenomes. RSCU values are represented on the *y*-axis, and families of synonymous codons and their respective amino acids are indicated on the *x*-axis. The synonymous codon families observed in *Psorophora albipes* (which had its RSCU graph enlarged to make the other graphs easier to read) are the same observed for the other mosquito species.

Disregarding the stop codons, there are a total of 3667 codons in use in the *Ad. squamipennis* mitogenome, followed by 3665 in *Ae. fluviatilis*, 3651 in *Cq. nigricans*, and 3650 in the *Ps. albipes* and *Ps. ferox* mitogenome. The relative synonymous codon usage (RSCU) showed that nearly all codons seem to be used on the five sequences, however the AGG (S) codon was not expressed at all. Furthermore, CUG (L) was expressed only in the *Ae. fluviatilis* mitogenome, and *Ps. albipes* did not express ACC (T) and CGC (R), likewise *Ps. ferox* did not express UCG (S) and CCG (P) (Figure 3). The RSCU analysis also showed that codons with adenine and/or thymine (uracil) in the latter position were highly expressed in contrast with other synonymous codons that express cytosine and/or guanine in the third position, the NNA/NNU RSCU value were often above 1 (Table S6).

Leucine (L) synthesized from the UUA codon was the most frequent amino acid in the five species (Figure 3), representing 5.09 on RSCU analysis for *Ps. albipes*, followed by 5.02 in *Ps. ferox*, 4.98 in both *Ae. fluviatilis* and *Ad. squamipennis*, and 4.97 in *Cq. nigricans*, further codons most frequently used were: arginine in CGA (R) type (average RSCU value 2.95) and serine in UCU (S) type (2.87). Leucine in the CUC type was the least frequent in *Ps. albipes* mitogenome (RSCU = 0.02), along with threonine (T) in ACG type and glycine in GGC type (RSCU = 0.02). The same leucine (CUC) was also the least frequent amino acid in *Ps. ferox* mitogenome (RSCU = 0.02), followed by glutamine in GAG (E) type (RSCU = 0.03). Threonine (ACG) was the least frequent codon in *Cq. nigricans* mitogenome (RSCU = 0.02) followed by proline in CCG type (RSCU = 0.03). Leucine in CUG and CUC type were the least expressed amino acids in *Ae. fluviatilis* (RSCU = 0.01 and 0.02, respectively). The CCG proline (P) had the lowest amino acid frequency in the *Ad. squamipennis* sequence, representing the RSCU value of 0.03 (CCG codon). In all sequences, the type codons NNC and NNG had RSCU value < 1, as observed in other studies of mitogenome sequencing of other mosquito and insect species [22,23,47,48,59,60,61].

### 3.3. Evolutive Analysis

**Figure 4:**
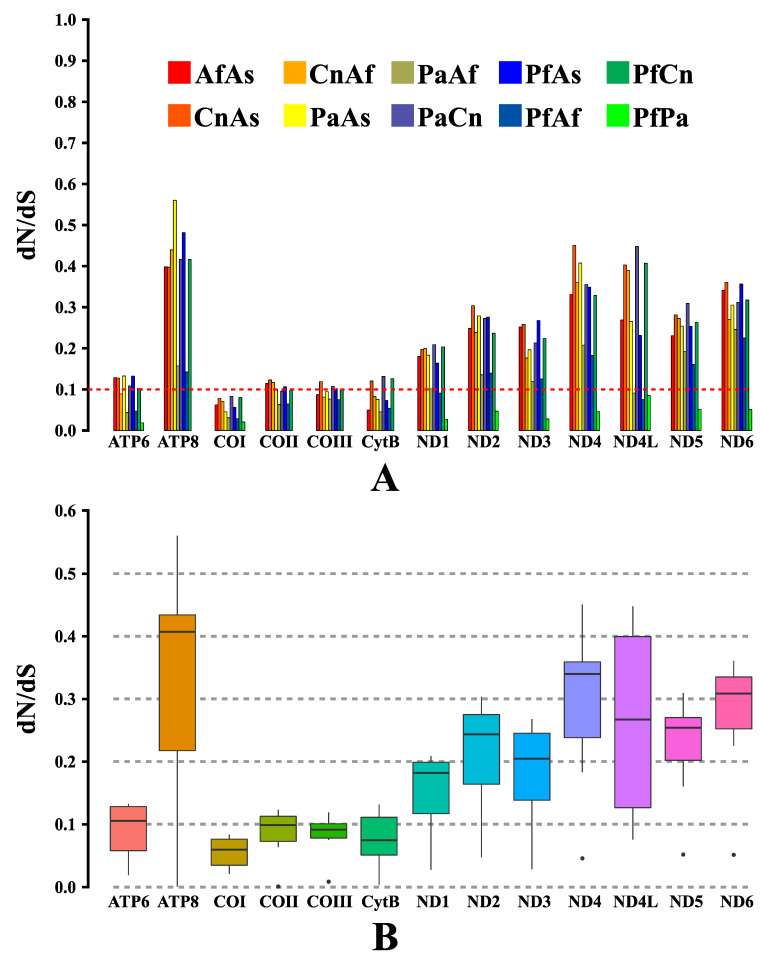
Proportions between rates of non-synonymous (*dN*) and synonymous (*dS*) nucleotide substitutions (*dN*/*dS*). (**A**) Bar chart for pairwise proportions of *dN*/*dS* for each of the mitochondrial subunits of the investigated species. (**B**) Box chart illustrating the averages for pairwise proportions of *dN*/*dS* for each of the mitochondrial subunits of the investigated species. In the graphs, the *dN*/*dS* ratios are plotted on the *y*-axis, and the PCGs are plotted on the *x*-axis.

In order to verify the evolutionary pressure acting on the different regions of the protein-coding mitogenomes obtained, based on the observation of ratios established between the non-synonymous and synonymous substitution rates (*dN*/*dS*), paired analyses were performed between the sequences corresponding to each PCG of the investigated species, with results plotted in two different graphical presentation models. The results obtained indicated that the different regions evaluated are evolving globally under the effect of negative (or purifying) pressure, with *dN*/*dS* values < 1 (Figure 4A,B), with ratios ranging from 0.0208 ± 0.0835 in *COI* to 0.0010 ± 0.5603 in *ATP8*, and thus, presenting the following order of influence of the purifying evolutionary pressure according to the averages obtained: *COI* < *CytB* < *COIII* < *COII* < *ATP6* < *ND1* < *ND3* < *ND2* < *ND5* < *ND4L* < *ND6* < *ND4* < *ATP8* (Figure 4B). Additionally, as reported in other studies, it was observed that the purifying evolutionary pressure was particularly stronger on PCGs belonging to mitochondrial complexes III (*CytB*) and IV (*COI*, *COII*, and *COIII*), in contrast to the regions of complex I (*NADH*), which, like *ATP8*, although with *dN*/*dS* rates < 1, showed less conservative evolutionary pressure restrictions, with relaxed purifying pressure and brief signs of positive evolutionary pressure (adaptive). The results obtained in this analysis still corroborate the pattern of heterogeneity between the evolutionary rates, although purifying, acting on different PCGs despite the taxonomic distance among the evaluated species [5,22,23].

**Figure 5:**
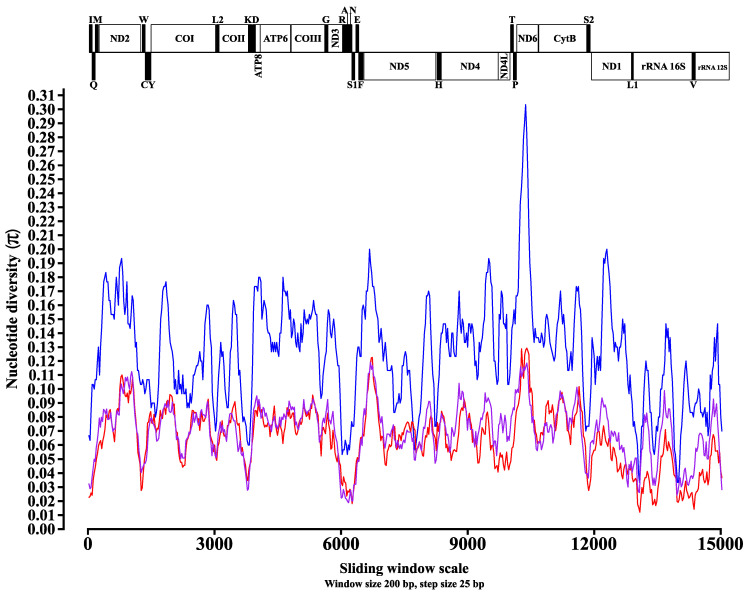
Nucleotide diversity (π) among the obtained mitogenomes in this study. Blue lines indicate nucleotide diversity values based on evaluation of sequences from the tribe Mansoniini, including *Cq. nigricans*. Red lines indicate nucleotide diversity values based on evaluation of sequences from the Aedini tribe, including *Ae. fluviatilis*, *Ps. albipes*, and *Ps. ferox*. The purple lines indicate the nucleotide diversity values based on the evaluation of the *Aedeomyia squamipennis* (Aedeomyiini) sequence compared to the Mansoniini and Aedini sequence groups. The π values were calculated from a 200 bp sliding window analysis in 25 bp steps and are plotted on the *y*-axis. The length values of the aligned sequences of the three evaluated groups are plotted on the *x*-axis. The limits of each gene are indicated in the representation above the graph: vertical black bars indicate tRNAs, and white rectangles indicate PCGs and rRNAs.

In addition to the *dN*/*dS* analysis, three analyses were performed in order to assess the nucleotide diversity between the mitochondrial sequences obtained in this study, together with other sequences of mosquitoes belonging to the Aedini and Mansoniini tribes (the same used for the reconstruction of the phylogenies presented in followed). The first analysis carried out considered the evaluation of nucleotide diversity among mitochondrial sequences of mosquitoes belonging to the Mansoniini tribe; the second analysis considered the evaluation of nucleotide diversity among the mitochondrial sequences of mosquitoes belonging to the Aedini tribe; and the third analysis considered the evaluation of nucleotide diversity among the *Ae. squamipennis*, the only representative of the Aedeomyiini tribe, together with the others evaluated and belonging to Aedini and Mansoniini. The results showed values of nucleotide diversity (π), considering the groups of evaluated sequences, ranging from 0.03 ± 0.3 in Mansoniini (Figure 5, blue line); 0.012 ± 0.128 in Aedini (Figure 5, red line); and 0.019 ± 0.118 evaluating *Ae. squamipennis* (Aedeomyiini) along with Aedini and Mansoniini (Figure 5, purple line).

The results obtained relate the highest rates of nucleotide diversity in the three groups of sequences evaluated to the regions acting in the protein coding of the analyzed mitogenomes, which it is observed that in at least four of these regions there were sites that reached the maximum diversity scores for each analysis, which are, in order of scoring: *ND6*, *ND5*, *ND2*, and *COI*. The results obtained, in this way, were similar to other previously published ones, when other species of mosquitoes were evaluated [22,23,48,62], highlighting, in addition, the high nucleotide diversity scores for the *COI* gene, used universally as one of the main molecular markers [63], in both analyses.

In mosquitoes, the proper identification of certain species is difficult due to the great morphological similarity they may present. Therefore, in certain situations, only an observation of internal structures, such as the male specimen’s genitalia, allows a reliable identification [64]. In this context, the alternative use of mitochondrial genes, such as *COI* or *ND5*, in the identification of cryptic species has shown satisfactory results [14,65,66,67]. Thus, the results obtained in this study, considering the investigated species, corroborate the use of PCGs as the most suitable regions to be used as molecular markers in the development of evolutionary studies of mosquito species.

### 3.4. Phylogenetic Analysis

The nucleotide sequence alignment using 13 PCGs from 48 taxa (five from this study, the remaining from *GenBank* and *EMBL* database), resulted in a nucleotide distance average of 0.16%, the values ranged from 0.03% to 0.29% (Table S7).

**Figure 6:**
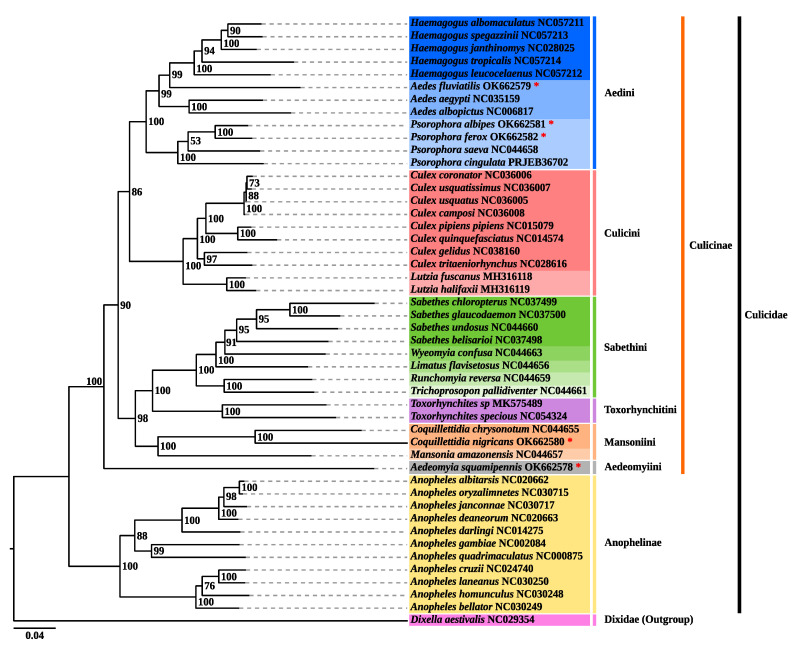
Phylogenetic reconstruction by maximum likelihood based on the 13 PCGs concatenated of the species sequenced in this study (marked with the red asterisk) and 42 other taxa with data available on the GenBank and EMBL databases. The values of support for bootstrapping (BPP) are shown on the left, in each node. Dash lines were drawn for viewing purpose.

**Figure 7:**
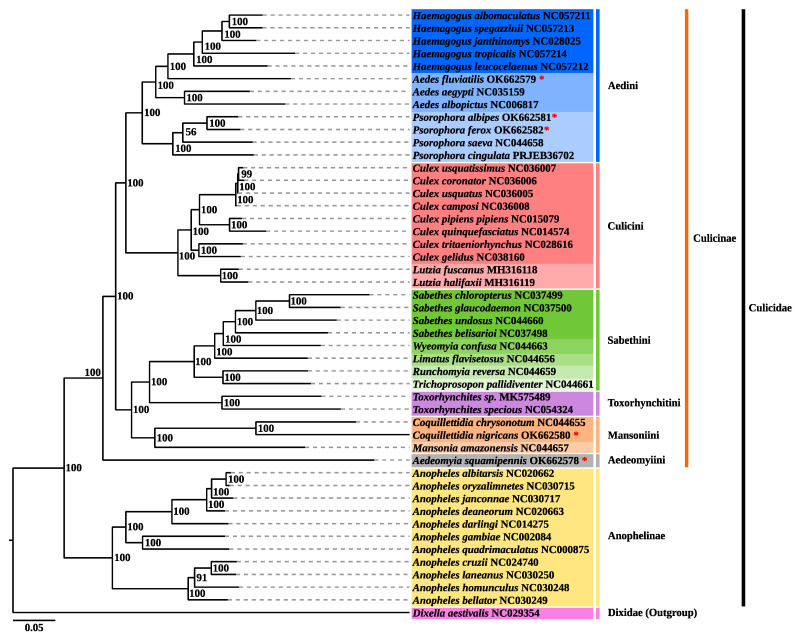
Phylogenetic reconstruction by Bayesian Inference based on the 13 PCGs concatenated of the species sequenced in this study (marked with the red asterisk) and 42 other taxa with data available on the GenBank and EMBL databases. The values of support Bayesian probabilities (BP) are shown on the left, in each node. Dash lines were drawn for viewing purpose.

The two methods of phylogenetic reconstruction (ML and BI) produced identical topologies with high internal anchoring values (Figure 6 and Figure 7), a monophyletic large group included 47 taxa corresponding to the Culicidae family, externally anchored by *Dixella aestivalis* (Diptera: Dixidae), defined by the automated method of midpoint [68]. The Culicidae family presented a topology with two well-supported clades (BPP/BP = 100%/100%), corresponding to the subfamilies Anophelinae and Culicinae. The Anophelinae clade, containing 11 species, is external in relation to Culicinae, and evidenced four subclades representing the subgenera *Nyssorhynchus*, *Cellia*, *Anopheles* and *Kertezia.* Our results corroborate the basal placement of subgenus *Kertezia* within the genus *Anopheles*, despite the no representativity of *Chagasia* genus, that so far is classified as most basal group [69,70,71]. New evolutionary evidence on temporal diversification has shown that radiation in the Culicinae subfamily is older than Anophelinae and that *Bironella* phylogenetic status is not stable [72].

The clade that represents the Culicinae subfamily comprises 36 taxa, and was structured into six subclades, representing the tribes Aedini, Culicini, Sabethini, Toxorhynchitini, Mansoniini, and Aedeomyiini (BPP/BP > 98%), on this arrangement: Aedeomyiini + [Mansoniini + (Toxorhynchitini + Sabethini)] + [Culicini + Aedini]. The Aedeomyiini, represented by the single newly sequenced *Ad. squamipennis* is placed as the most basal taxa in the Culicinae subfamily anchoring externally all remaining Culinae tribes, strongly supported by the BPP/BP = 100%/100% values. These results differs from those obtained by Reindenbach et al. [11] who placed *Aedeomyia + Toxorhynchites* as an intermediary taxa, between Sabethini and Aedini tribes in a phylogenetic analysis using six nuclear protein genes. Evolutionary studies with the incomplete mitogenome sequence had clustered *Ae*. *squamipennis* with either *Uranotaenia* or *Toxorhynchites* genera, but more than that, it suggested that *Ad. squamipennis* is the earliest divergent taxa in the Culicinae subfamily, with speciation process around 150 million years ago (MYA) [73]. Indeed, the *Aedeomyia* genus (Theobald, 1901) is promptly distinguished by morphological aspects, and it is thought to be ancient, but extremely specialized taxa that arose in the Old World and spread to some continents, being *Ad. squamipennis* the single species occurring in the New World. The evolutionary time scale and the geographic boundaries may have contributed to this suggested phylogenetic distance and constitutes an object to further studies [73,74]. Phylogeny inferred from complete mitogenome has producing consistent and more accurate results for evolutionary and taxonomic studies [23], therefore, our results sheds light on the evolutionary history of Aedeomyiini tribe but demands sampling efforts to build a solid molecular taxonomy.

The phylogenetic analysis of *Cq.* (*Rhy*.) *nigricans* resulted in its placement in the Mansoniini tribe (BPP/BP = 100%/100%) and *Coquilletidia* genus (BPP/BP = 100%/100%), related to *Mansonia* genus (BPP/BP = 100%/100%), in accordance with the current taxonomic classification [75,76]. Regarding the external relationships, our results generate a subclade Mansoniini + [Toxorhynchitini + Sabethini] (BPP/BP = 98%/100%). Phylogenetic relationships of the Mansoniini are widely accepted to be monophyletic, supported by morphological and bionomic aspects, mainly synapomorphies of immature stages [3]. Moreover, these findings are in accordance with recent evolutionary studies that confirmed the monophyletic status of the tribe, and suggested that Mansoniini is a sister clade of Sabethini tribe that arose nearly 85 MYA [72]. On the other hand, our results contrast with former analysis that related Mansoniini to Aedini tribe, but the non-aedini species were missampling and the target for phylogenetic analysis were different [11,77].

In this study, three representatives of the Aedini tribe were investigated: *Ae.* (*Grc*.) *fluviatilis*, *Ps.* (*Jan*.) *albipes*, and *Ps.* (*Jan*.) *ferox*. The phylogeny obtained presented a well-supported clade corresponding to the Aedini tribe (BPP/BP = 100%/100%). This group included three closely related taxa, corresponding to *Haemagogus*, *Aedes*, and *Psorophora* genera, recovered as sister group to Culicini tribe (BPP/BP = 100%/100%), as already suggested by other studies [11,23,72]. *Psorophora* had basal positioning as already observed by other authors [11,72]. Molecular evolutionary studies suggested a diversification between these tribes around 130 MYA, in the Cretaceous period [72].

The newly sequenced *Ae. fluviatilis* was placed as a sister group to the *Haemagogus* clade (BPP/BP = 98%/100%), a topology possibly induced by a missrepresentation of *Aedes* species. For instance the average of nucleotide distance in tribe level was 0.14, and very similar values were obtained when compared *Ae. fluviatilis* to the other genera (*Aedes*, *Psorophora* and *Haemagogus*). *Ae. fluviatilis* is an anthropophilic mosquito commonly found in urban areas, distributed in the Neotropical region and is considered a potential vector of yellow fever virus. Given its epidemiological potential, recent phylogenetic studies with incomplete mitogenome sequence had found that *Ae. fluviatilis* is an ancient diverged species regarding the *Aedes* genus, but with no phyletic stability [72].

The Aedini topology suggests that *Ae. fluviatilis* is an independent group. In fact there is a strong taxonomic trend that elevates the subgenus *Georgecraigius* to genus status [78]. Massive morphological data were extensively studied and led to this conclusion, therefore, *Georgecraigius* would comprise subgenera *Georgecraigius* and *Horsfallius*, with three species composing [75,79]. In the studies of Reinert, the *Georgecraigius* genus relates to an Australasian genus, *Patmarksia.* Later, in order to make Aedini taxonomy treatment more parsimonious, Wilkerson et al. [80] reanalyzed the whole set of morphological data and proposed simplified generic designations, among which *Georgecraigius* was restored to subgenus level. Aedini is the largest tribe in the Culicidae family and there is an urgent need for much broader studies to reconstruct the natural history of the group.

The topology obtained for the newly sequenced *Psorophora* species clustered *Ps. albipes* + *Ps. ferox* on a subclade that here represents the *Janthinosoma* subgenus (BPP/BP = 100%/100%), on the monophyletic genus *Psorophora* (BPP/BP = 100%/100%), and Aedini tribe (BPP/BP = 100%/100%), recovered as sister group to *Aedes* + *Haemagogus* clade, as already found by Reinert et al. [75,78,79] and Soghigian et al. [81]. The generic rank retrieved in this analysis for Aedini tribe requires further studies for better resolution, as demonstrated by the *Janthinosoma* + *Psorophora* (BPP/BP = 53%/56%) subclade. In the study carried out by Silva et al. [72] the incomplete mitogenome sequence of *Ps. albipes* was assessed and the molecular evolutionary findings clustered *Janthinosoma* with *Grabhamia*, but the analysis included only two members of the genus, and no *Psorophora* (*Pso*.) species.

Morphological and bionomical aspects were targeted for phylogenetic reconstruction of *Psorophora* genus in earlier studies [77], and molecular evidence aggregated more value to this taxonomic classification [81]. Nonetheless, the resort of a unique tool, such as morphology, may lead to results that do not always reflect the natural history of the species. Cladistic analysis using morphology recovered the monophyly of the subgenera, although some internal polytomies on the *Janthinosoma* and *Grabhamia* were recorded [82].

The investigation over the mitochondrial genome of five species of mosquitoes occurring in the Brazilian Amazon region allowed us to obtain important information about the taxonomy and evolutionary biology of the Culicidae. Classical taxonomy based on the morphology and bionomics of the species is undoubtedly the main path to classification process, nevertheless, such method has its flaws, especially when it comes to a taxa as diverse and ancient as the Culicidae family: morphological and bionomical characteristics may arise and vanish in the same group over time. The advent of molecular tools has allowing deeper investigation on the taxonomic relationships between these organisms, recent studies has providing knowledge on the natural relatedness, biogeographical and ancestry. In this sense, it is important to highlight that mitochondrial characterization studies and the consequent inclusion of more Culicidae taxa in public databases will support more comprehensive phylogenetic reconstruction analyses and elucidate taxonomic proposals.

## 4. Conclusions

The aim of this study was to enrich knowledge on the molecular aspects and to propose a phylogenetic reconstruction of the Culicidae family based on mtDNA. This is the first description of the complete mitochondrial DNA of *Ae.* (*Grg*.) *fluviatilis*, *Ad. squaminpennis*, *Cq.* (*Rhy*.) *nigricans*, *Ps*. (*Jan*.) *albipes* and *Ps*. (*Jan*.) *ferox*. All mitogenomes evaluated were similar to the mtDNA molecule pattern for Culicidae family: 37 subunits subdivided in 13 PCGs, two rRNAs and 22 tRNAs. The analyses of the individual genes showed that *COI* is the most preserved subunit and more suitable for taxonomic purposes and *ATP8* is the less conservative gene, the 13 PCGs combined demonstrated to be a more complete object of study for both taxonomic and phylogenetic analyses. ML and BI inference yielded identical topologies where *Ad. squamipennis* was placed as the most external lineage from Culicinae subfamily; *Cq. nigricans* related to *Cq. chrysonotum*, both related to *Mansonia; Ae. fluviatilis* positioned in Aedini tribe but as a sister clade to *Aedes* (Stg.) spp. which clearly demonstrated the necessity for more representative studies within the genus; both *Ps. albipes* and *Ps. ferox* clustered together as the *Janthinosoma* group on *Psorophora* subclade, this the first analysis with complete mtDNA emcompassing all subgenera of the genus. All phylogenetic results confirm the current taxonomic proposition for the studied tribes. These finding will support future hypotheses on taxonomic and evolutionary traits for the Culicidae family.

## Figures and Tables

**Table 1:** Collection information on the evaluated species.

Species	ID-Code	Collected	Used	Sex	Collection Site	State	Geographical Coordinates	Year
*Ad. squamipennis*	AR_867100	6	5	♀	Altamira	Pará	−3.203333, −52.206389	2017
*Ae. fluviatilis*	AR_866516	13	5	♀	Canaã dos Carajás	Pará	−6.496944, −49.878333	2019
*Cq. nigricans*	AR_850512	45	5	♀	Altamira	Pará	−3.203333, −52.206389	2017
*Ps. albipes*	AR_859743	30	5	♀	Belém	Pará	−1.495795, −48.454260	2017
*Ps. ferox*	AR_849712	14	5	♀	Santa Bárbara	Pará	−1.209000, −48.271722	2017

**Table 2:** Taxonomic confirmation of evaluated species based on the barcode sequence of COI genes. Access codes from species presented below may be consulted on the NCBI database.

Species Evaluated	Nucleotide Identity (%)—COI (Barcode DNA)				
	*Ad. squamipennis* AF417729	*Oc. fluviatilis* KF314736	*Cq. nigricans* KM593033	*Ps. albipes* MN997524	*Ps. ferox* MN997516
*Ad. squamipennis*	98.99	-	-	-	-
*Ae. fluviatilis*	-	98.78	-	-	-
*Cq. nigricans*	-	-	99.85	-	-
*Ps. albipes*	-	-	-	95.14	-
*Ps. ferox*	-	-	-	-	99.7

**Table 3:** General metrics of the mitogenomes obtained in this study.

Species	Size (bp)	A%	T%	C%	G%	AT%	AT-Skew	GC-Skew
Whole mitogenome								
*Aedeomyia squamipennis*	15,087	40.3	37.9	13.0	8.8	78.2	0.0307	−0.1927
*Aedes fluviatilis*	14,910	40.4	37.1	13.3	9.2	77.5	0.0426	−0.1822
*Coquillettidia nigricans*	14,789	40.0	38.9	13.3	7.8	78.9	0.0139	−0.2607
*Psorophora albipes*	15,137	39.3	38.6	13.1	9.0	77.9	0.0090	−0.1855
*Psorophora ferox*	15,307	39.1	38.5	13.2	9.1	77.6	0.0068	−0.1839
PCGs								
*Aedeomyia squamipennis*	11,170	32.6	44.1	11.3	12.0	76.7	−0.1499	0.0300
*Aedes fluviatilis*	11,216	32.6	43.3	11.8	12.2	75.9	−0.1410	0.0167
*Coquillettidia nigricans*	11,357	33.4	44.4	10.8	11.3	77.8	−0.1414	0.0226
*Psorophora albipes*	11,269	32.5	44.1	11.4	12.0	76.6	−0.1514	0.0256
*Psorophora ferox*	11,270	32.1	44.0	11.6	12.4	76.1	−0.1564	0.0333
tRNAs								
*Aedeomyia squamipennis*	1470	40.8	38.9	8.9	11.4	79.7	0.0238	0.1232
*Aedes fluviatilis*	1486	40.4	39.4	8.7	11.4	79.8	0.0125	0.1343
*Coquillettidia nigricans*	1479	41.3	39.7	8.0	11.0	81.0	0.0198	0.1579
*Psorophora albipes*	1493	39.5	39.2	9.2	12.0	78.7	0.0038	0.1321
*Psorophora ferox*	1494	40.1	39.3	8.8	11.8	79.4	0.0101	0.1456
rRNAs								
*Aedeomyia squamipennis*	2160	38.9	43.8	6.0	11.3	82.7	−0.0593	0.3064
*Aedes fluviatilis*	2151	39.1	43.6	6.1	11.2	82.7	−0.0544	0.2948
*Coquillettidia nigricans*	2097	39.6	42.7	5.4	12.2	82.3	−0.0377	0.3864
*Psorophora albipes*	2154	39.3	42.7	6.3	11.7	82.0	−0.0415	0.3000
*Psorophora ferox*	2160	39.4	42.6	6.3	11.7	82.0	−0.0390	0.3000

## Data Availability

All data generated during this study are available as tables and figures included in this published article and its Supplementary Information files. The GenBank database accession numbers for the four mitochondrial genomes sequenced in this study are OK662581 (*Ps. albipes*), OK662582 (*Ps. ferox*), OK662579 (*Ae. fluviatilis*), OK662580 (*Cq. nigricans*), and OK662578 (*Ad. squamipennis*).

## Supplementary Materials

The following content are available online at https://www.mdpi.com/article/10.3390/genes12121983/s1, Figure S1: Secondary structures of *Ps. albipes* tRNAs, Figure S2: Secondary structures of *Ps. ferox* tRNAs, Figure S3: Secondary structures of *Ae. fluviatilis* tRNAs, Figure S4: Secondary structures of *Cq. nigricans* tRNAs, Figure S5: Secondary structures of *Ad. squamipennis* tRNAs, Table S1: Nucleotide composition of the mtDNA of *Ps. albipes*, Table S2: Nucleotide composition of the mtDNA of *Ps. ferox*, Table S3: Nucleotide composition of the mtDNA of *Ae. fluviatilis*, Table S4: Nucleotide composition of the mtDNA of *Cq. nigricans*, Table S5: Nucleotide composition of the mtDNA of *Ad. squamipennis*, Table S6: RSCU metrics of obtained sequences, Table S7: Nucleotide distance matrix of taxa used in phylogeny reconstruction analysis.
