# Supplementary material for: First Description of the Mitogenome and Phylogeny of Culicinae Species from the Amazon Region
Source: Genes (Basel). 2021 Dec 14;12(12):1983. doi: 10.3390/genes12121983 (PMC8701802; doi:10.3390/genes12121983)

Aedeomyia squamipennis

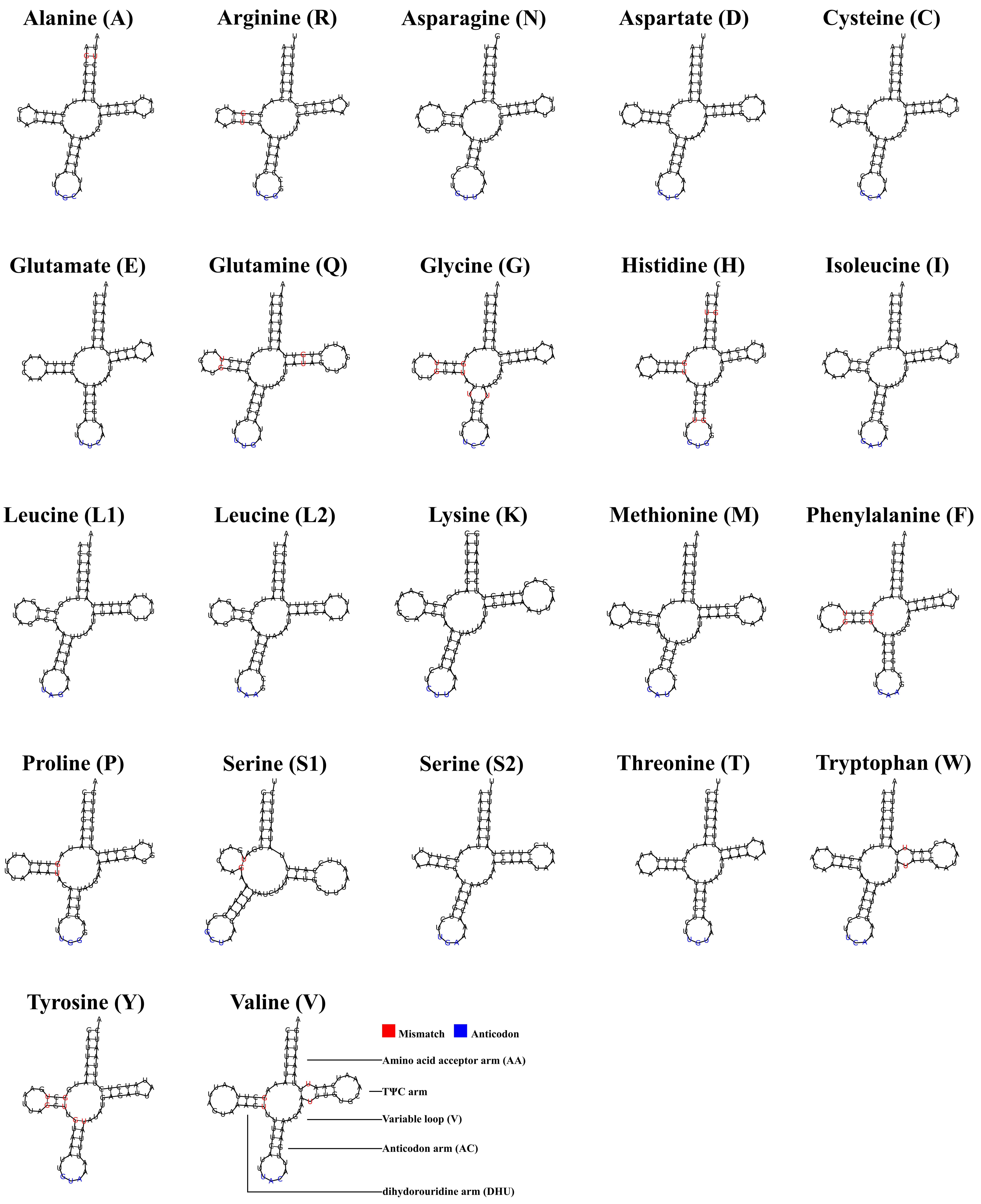

Supplement: Supplementary file 1 [file genes-12-01983-s001.zip › figure_s5.pdf]
